# Evaluation of the dimensional changes in the mandible, condyles, and the temporomandibular joint following skeletal class III treatment with chin cup and bonded maxillary bite block using low-dose computed tomography: A single-center, randomized controlled trial

**DOI:** 10.12688/f1000research.130941.1

**Published:** 2023-03-13

**Authors:** Amr H. Husson, Ahmad S. Burhan, Mohammad Younis Hajeer, Fehmieh R. Nawaya

**Affiliations:** 1 Department of Orthodontics, University of Damascus Faculty of Dentistry, Damascus, Damascus, Syria; 2 Department of Pediatric Dentistry, Faculty of Dentistry, Syrian Private University, Damascus countryside, Syria

**Keywords:** Skeletal Class III malocclusion, chin cup, low-dose computed tomography, lose-dose CT, volumetric assessment

## Abstract

**Background: **Insufficient evidence regarding the effects of chincup therapy on the mandibular dimensions and temporomandibular joint (TMJ) structures requires high-quality studies using three-dimensional (3D) imaging. This trial aimed to evaluate the 3D changes in the mandible, condyles, and glenoid fossa after chin cup therapy for skeletal Class III children compared to untreated controls.

**Methods:** A 2-arm parallel-group randomized controlled trial on 38 prognathic children (21 boys and 17 girls), with mean ages 6.63±0.84 years. Patients were recruited and randomized into two equal groups; the experimental group (CC) was treated with occipital-traction chin cups in conjunction with bonded maxillary bite blocks. No treatment was provided in the control group (CON). Low-dose CT images were acquired before (T1) and after achieving  (2-4 mm) positive overjet (T2), and after 16 months apart in both groups. The outcome measures of the condyle-mandibular 3D distances, the condyles-glenoid fossa postional changes, and the quantitative displacement parameters of superimposed 3D models were compared statistically. Paired- and two-sample t-tests were used for intra- and inter-group comparisons, respectively.

**Results:** Overall, 35 patients (18 and 17 in the CC and the CON groups, respetively) were enrolled in the statistical analysis. The mean mandibular and condylar volumes increased significantly by 777.24 mm ^3^ and 1,221.62 mm ^3^, 94.57 mm ^3^, and 132.54 mm ^3^ in the CC and CON groups, respectively. No statistically significant differences were observed between the groups regarding the volumes, superficial areas, and linear changes of the mandible and condyles, and part analysis measurements, except the changes of the relative sagittal and vertical positions of condyles, glenoid fossa, and posterior joint space, which were significantly smaller in the CC group (p<0.05) than the CON group.

**Conclusions:** The chin cup did not affect the mandibular dimensions. Its primary action was confined to the condyles and the TMJ internal dimensions.

**Clinicaltrials.gov registration**: NCT05350306 (28/04/2022).

## Introduction

The efficacy of the chin cup in controlling mandibular growth remains a controversial matter. ^1^^,^^2^ Findings in some clinical studies support retardation or restriction of mandibular growth, ^3^^–^^5^ but others show the opposite. ^6^^,^^7^ Still, other studies highlight only changes in shape and redirection of mandibular growth. ^8^^,^^9^ The conclusion of Liu's ^2^ systematic review indicated insufficient data to judge the efficacy of the chin cup on mandibular inhibition. A recent systematic review by Chatzoudi ^1^ concluded that more high-quality studies with proper methodology, untreated control groups, and reliable measurements are needed. Moreover, the supposed changes in the temporomandibular joint (TMJ) due to the retraction force applied by a chin cup is not yet clear. Zurfluh ^10^ stated that the low-quality data from past studies hindered pointing out clear statements regarding the effects of chin-cup treatment on TMJ. Nevertheless, TMJ changes associated with this treatment need to be evaluated.

Previously, mandibular adaptation to chin cup force has been evaluated using two-dimensional (2D) lateral cephalograms or panoramic radiographs. However, the information provided by these techniques is limited in evaluating the condyles and mandibular ramus. ^11^ Therefore, the three-dimensional (3D) analysis using 3D computed tomography (CT) or cone-beam CT (CBCT) has become important. ^12^ Although CBCT uses relatively low radiation compared to conventional CT, the accuracy of this technique is still doubtful in detecting condylar changes. ^13^^,^^14^ Low-dose CT is an alternative reliable procedure with an effective dose approximately equal to traditional radiographs. ^15^ This technique has been used in some clinical studies. ^16^^,^^17^

Histologically, experimental animal studies have demonstrated a reduction in the width of the prechondroblastic zone and a decrease in the cellularity of the condylar head after chin cup therapy. ^18^ In this respect, investigating the volumetric changes in condyles may be useful.

To our knowledge, no study has investigated the volumetric changes of the mandible and condyles after early chin cup therapy. This randomized controlled trial aimed to evaluate the changes in the mandible, condyles, and glenoid fossa following the chincup therapy of Class III malocclusion patients in comparison with those changes in a control group of untreated patients. It aimed to test the null hypotheses that no significant differences existed between the chincup and the control group concerning the changes in the mandible, the condyles, and the glenoid fossa.

## Methods

### Study design and registration

The research was designed as a single-center, 2-arm, parallel-group randomized controlled trial with a 1:1 allocation ratio and guided by the CONSORT statement. ^19^ The study was conducted in the Department of Orthodontics at the University of Damascus Dental School between January 2019 to January 2022. This study was approved by the Regional Ethical Committee on Research of the Damascus University, Faculty of Dentistry, Syria (UDDS-2788-2016PG) on January 11, 2019. The study was registered at ClinicalTrails.gov (Identifier: NCT05350306) on 28 April, 2022 after the onset of the study. Retrospective registration was done as the regulations at the Univeristy of Damascus do not oblige researchers to register their trial protocols before the commencement of their research work. However, this protocol was registered retrospectively to publish the trial's results. This article is reported in line with the CONSORT guidelines. ^44^

### Sample size estimation

Sample estimation was calculated using Minitab ^®^ Version 18 (Minitab Inc., State College, Pennsylvania, USA). An independent t-test, statistical power of 85%, and a significance level of 0.05 were among the assumptions for this calculation. In total, 17 children were required in each group to detect a 1 mm significant difference in total mandibular length (Co-Gn) based on the standard deviation of this measurement for a previous study (0.92). ^20^ Four patients were added to the experimental and control groups (2 patients in each group) to compensate for potential dropouts.

### Patient recruitment

**Figure 1.**
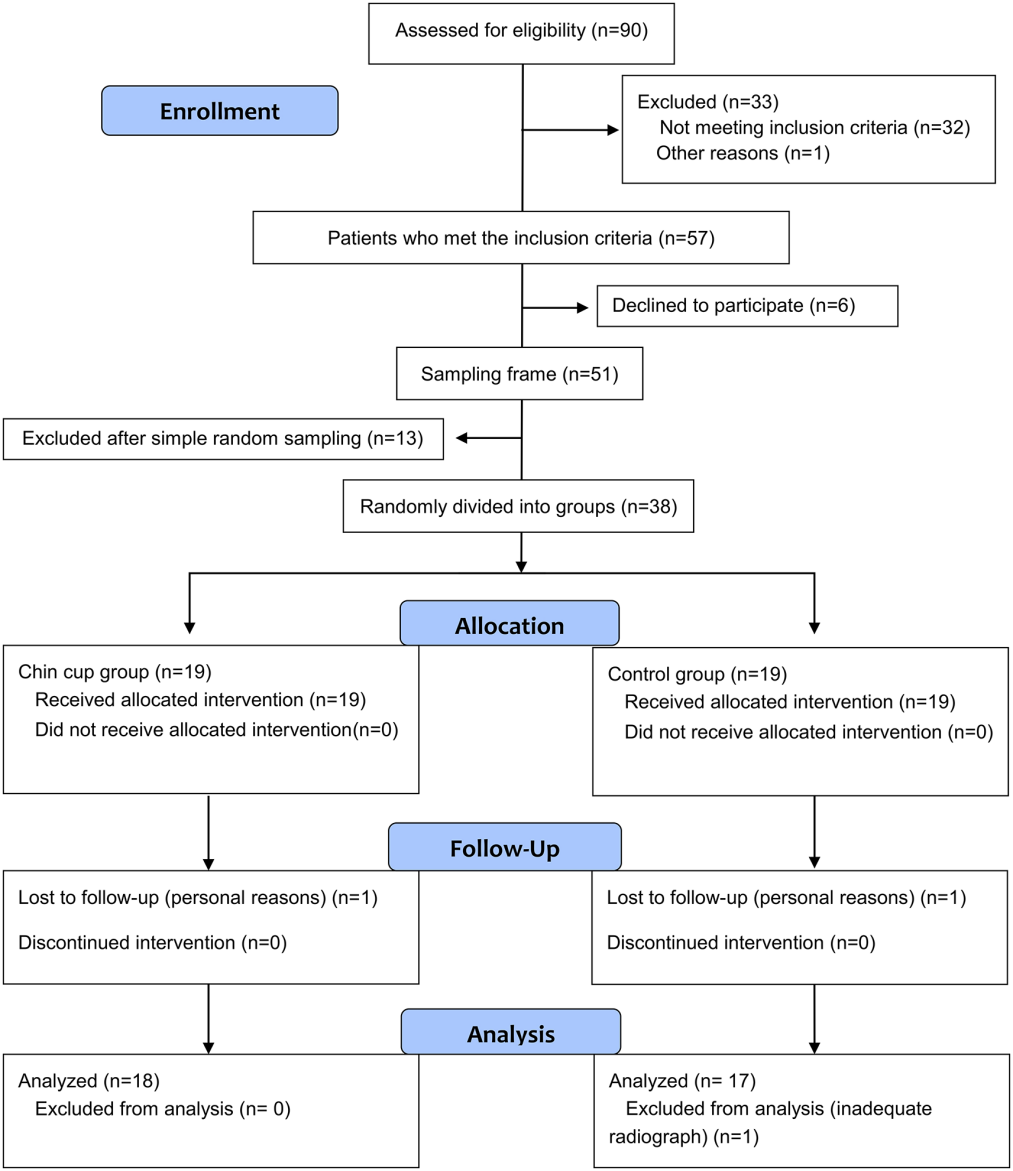
CONSORT flow diagram of patients’ recruitment, follow-up and entry into data analysis.

A preliminary screening was performed of schoolchildren during the period from April 2019 to May 2019. Ten public and private schools were randomly selected from the database of all primary schools in Damascus city, Syria. Intra- and extra-oral clinical examination was performed by the principal investigator (A.H.H.) for 1700 children with an average age of 7 years (range: 6-8 years), including 767 female and 933 male children at the medical clinics of the selected schools. Initially, 90 children with class III malocclusion (52 boys, 43 girls) were expected to be suitable for inclusion in the trial. A formal invitation letter was sent to the parents of candidate children asking them to visit the Department of Orthodontics at the University of Damascus for further analysis and to assess the need for early correction of the deformity. All invited parents came for this in-depth assessment. The following inclusion criteria were used: age between 6 and 8, edge-to-edge incisor relationship or anterior crossbite, class III relationships at the permanent first molars or mesial-step relationships at primary second molars, absence of discrepancy between the centric relation and the maximum intercuspation position, short or normal-face pattern, normal or deep overbite, and no temporomandibular joint disorders or craniofacial anomalies. The radiological inclusion criteria were (1) mild to moderate mandibular prognathism (SNB > 80°, and 0 ≤ANB≥4) and (2) normal or horizontal growth pattern (Bjork’s sum ≤396° ±5°). Fifty seven patients met the inclusion criteria. Information sheets were given to parents of eligible patients. The need for two low-dose CT images, and the possibility of postponing treatment if the child fell into the control group were elaborately explained before taking their written informed consents. Six parents refused to participate in this trial. Therefore the resultant sampling frame included 51 patients. Simple random sampling was used to select 38 patients for this trial who were then assigned randomly to the chin cup and the control groups. The CONSORT flow diagram of patients’ recruitment, follow-up, and entry into data analysis is given in Figure 1.

### Randomization and allocation concealment

An online randomization service ( www.randomizer.org) was used to perform simple randomization with an allocation ratio of 1:1. The allocation sequence was concealed from the researcher (AH) using sequentially numbered, opaque, and sealed envelopes, which were opened after patients completed all baseline assessments. Blinding of either patient or operator was not possible. Blinding was applied for the outcome assessment only. After the mandibular mask was constructed by one of the co-authors (MYH), the row image was resliced by cutting the region of the overjet to blind the other investigator (AH), who completed the extraction of results.

**Interventions**

*Experimental group: Chin-cup group*

**Figure 2.**
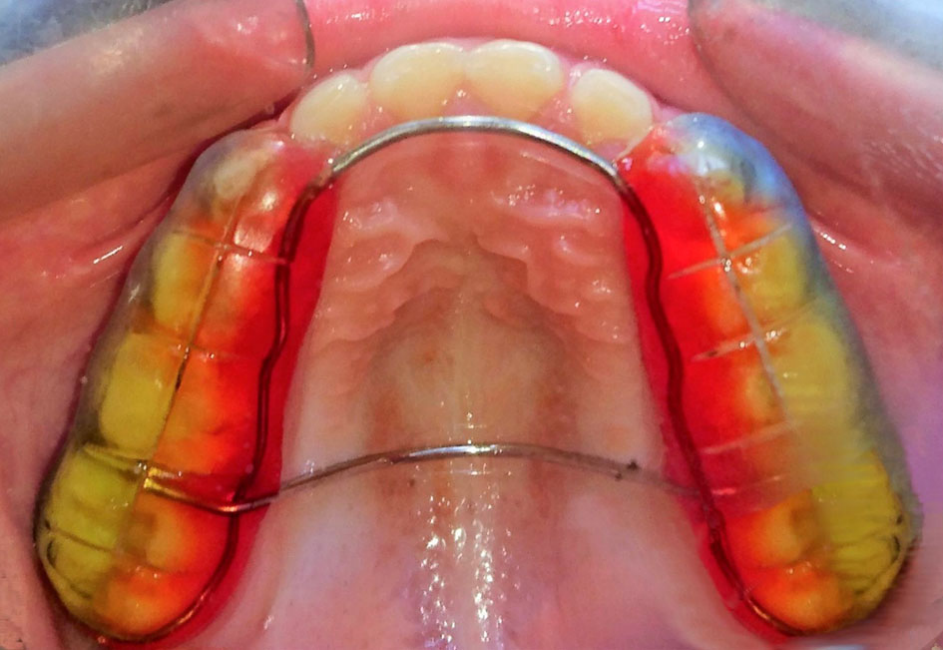
The bonded maxillary bite block used intra-orally in conjunction with the chincup appliance.

Each patient in the treatment group received a bonded maxillary bite block and an occipital chin cup ( Figure 2 shows the bite block). The splint consisted of a 2 mm posterior acrylic cap reinforced by a metal framework. The traction force on the mandible by the chin cup was 400–500 g at each side, in the direction of the condyles. ^8^ Patients were instructed to wear the appliance for 14 hours per day. The true overjet and overbite were measured monthly, subtraction the effects of the bite block on these variables based on the assumption that every 1 mm of the thickness of the posterior bite block increases the overjet by an average of 1.3 mm and decreases the overbite an average of 2 mm. ^21^ After achieving 1 mm overjet and 2 mm overbite, the acrylic splint was removed. Then, the chin cup was used with a light force for approximately 8 hours per day as a retainer. The active treatment was considered complete when the following two conditions were met: a positive overjet of 2 to 4 mm, and an active treatment time of at least 16 months.

### Control group

After acquiring low-dose CT images, the patients allocated to the control group received no clinical intervention. They were recalled 16 months after registration for collection of the second low-dose CT image records.

### Outcome measures

**Computed tomography acquisition**

A multiplanar spiral CT machine (Philips brilliance 64, Philips Medical Systems, Best, the Netherlands) was used to obtain pre- (T1) and post-treatment (T2) CT images. The CT scans were performed at 80 kV and 100 mAs, one pitch, 2.5 mGy (CTDIvol), and a 1.25 mm slice thickness. ^17^ The patients were seated in a supine position with their Frankfort horizontal (FH) plane perpendicular to the floor and their teeth in maximum intercuspation. Once the low-dose CT images were stored in the Digital Imaging and Communications in Medicine (DICOM) format, they were transferred into MIMICS 21.0 software (Materialise, Leuven, Belgium) to create 3D volumetric mandibular models.

**Mandible and condyles reconstruction**

Each image was resliced to make the head orientation uniform along the midsagittal and Frankfort horizontal planes. The basion, crista galli, and glabella landmarks defined the midsagittal plane. The Frankfort horizontal plane was defined by the right and left orbitale landmarks and the right porion landmark. ^22^

**Figure 3.**
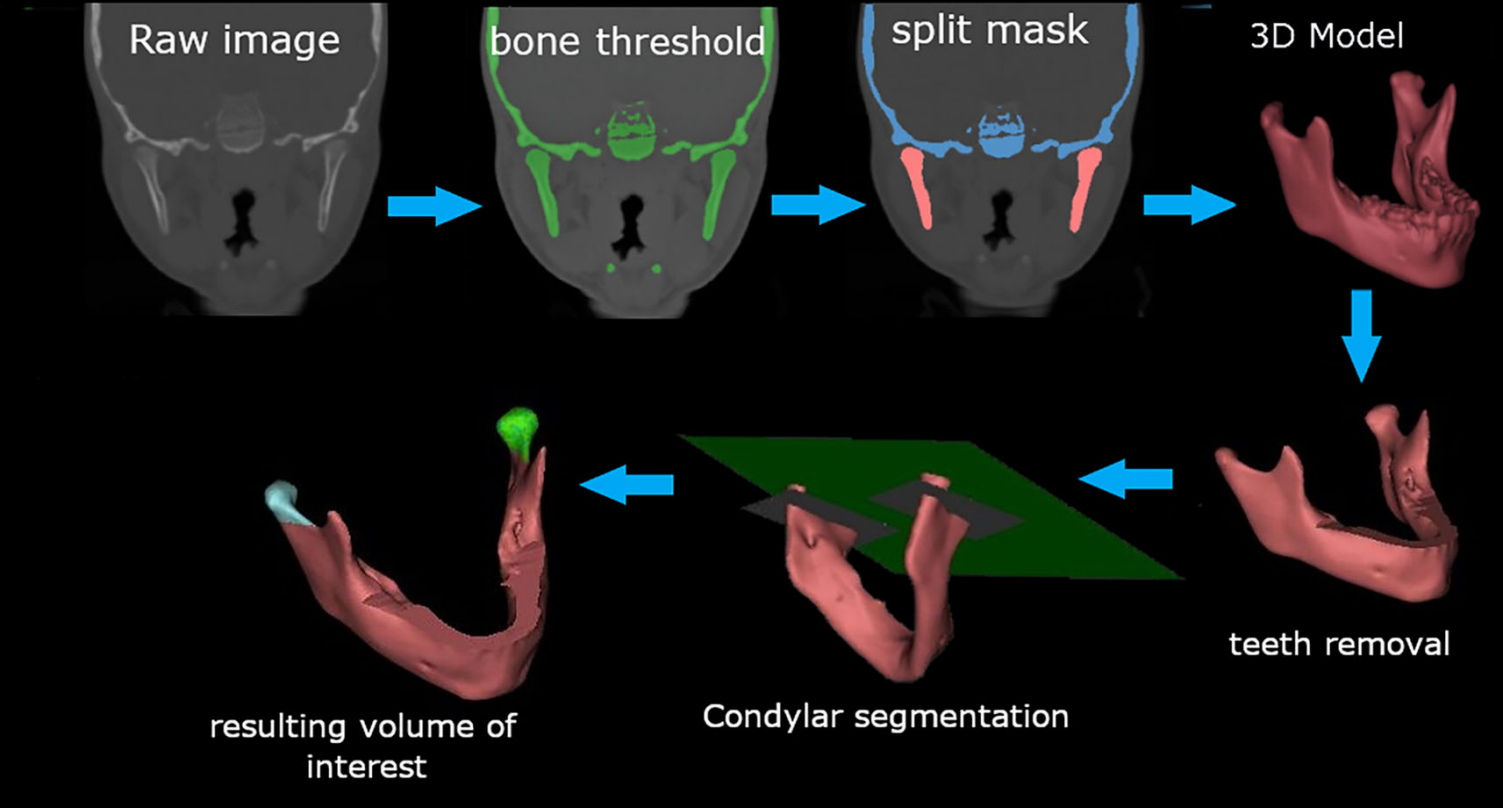
Workflow of the image processing procedures.

The image processing procedures are shown in Figure 3. Briefly, a bony mask was created using a global threshold value of 226 to 2976 Hounsfield units [HU] for the software algorithm. Semiautomatic segmentation using a split mask tool was utilized to isolate the mandibular bone. The mandible mask was rendered in a high-quality 3D model. Due to the differences in the lower permanent molar eruption, the crowns of the teeth were removed by a plane passing through 1 mm inferior to the alveolar bone and 10 mm distal to the second primary molars. Finally, the condyles were cut at neck level using a plane parallel to the Frankfort horizontal plane at the most inferior part of the sigmoid notch, and the post-treatment cut was parallel to the first cut. ^23^ The condyle and mandible volumes and surface sizes were automatically calculated in mm ^3^ and mm ^2^, respectively.

**Linear and angular measurements**

A new template was created from the Measure and Analyze tool of Mimics™, which was used to define the anatomical landmarks and planes and produce the desired measurements ( Tables 1 and 2). ^22^^,^^24^^,^^25^ Ikeda and Kawamura suggested that the condylar and glenoid fossa landmarks were made on the corrected sagittal view. ^26^ After identifying the landmarks, the software automatically calculated the distances and the angles. Then, the data was exported in.cvs format.

**Table 1.** Definitions of the anatomical landmarks and Reference planes. *

Anatomical landmarks				
	Landmark	Abbreviation	Bilateral	Description
Mandibular landmark	Gonion	Go	Yes	Most posterior and inferior point on the mandibular ramus
	Gnathion	Gn	No	The most anterior and inferior border of the chin in the mandibular midline
Condyle	Condylar superior	CS	Yes	Most superior point and the midpoint of the condyle
	Condylar posterior	CP	Yes	Most posterior point of condyle
	Condylar anterior	CA	Yes	Most anterior point of condyle
Mandibular fossa	Fossa superior	FS	Yes	Most superior point of mandibular fossa
	Fossa posterior	FP	Yes	Most anterior point on the posterior wall of mandibular fossa
	Fossa anterior	FA	Yes	Most posterior point on the anterior wall of mandibular fossa
Reference planes	Frankfort horizontal plane	FH	No	Defined by the right and left orbitale landmarks and the right porion landmark
	Perpendicular plane	Y	Yes	Normal plane to FH and midsagittal plane at porion landmark

* Definitions of these measurement are taken from: Mavreas and Athanasiou, ^24^ Alhammadi *et al*., ^22^ and Celikoglu *et al*. ^25^

**Table 2.** Definitions of the linear and angular measurements. *

	Landmark	Abbreviation	Bilateral	Description
Mandible	Mandibular body	Go-Gn	Yes	Distance between Go and Gn
	Ramal height	Co-Go	Yes	Distance between Co and Go
	Total body length	Co-Gn	Yes	Distance between Co and Gn
	Mandibular angle	Co-Go-Gn	Yes	Angle between Co, Go and Gn
Condyle	Vertical position of condyle	CP-FH	Yes	Distance of point CP from FH
	Sagittal position of condyle	CP-Y	Yes	Distance of point CP from Y
Mandibular fossa	Vertical position of mandibular fossa	FP-FH	Yes	Distance of point FP from FH
	Sagittal position of mandibular fossa	FP-Y	Yes	Distance of point FP from Y
Condyle-fossa relationship	Anterior joint space	AJS	Yes	Distance between CA and FA
	Superior joint space	SJS	Yes	Distance between CS and FS
	Posterior joint space	PJS	Yes	Distance between CP and FP

* Modified from: Mavreas and Athanasiou, ^24^ Alhammadi *et al*., ^22^ and Celikoglu *et al*. ^25^

**3D mandibular regional superimposition and comparison analysis**

**Figure 4.**
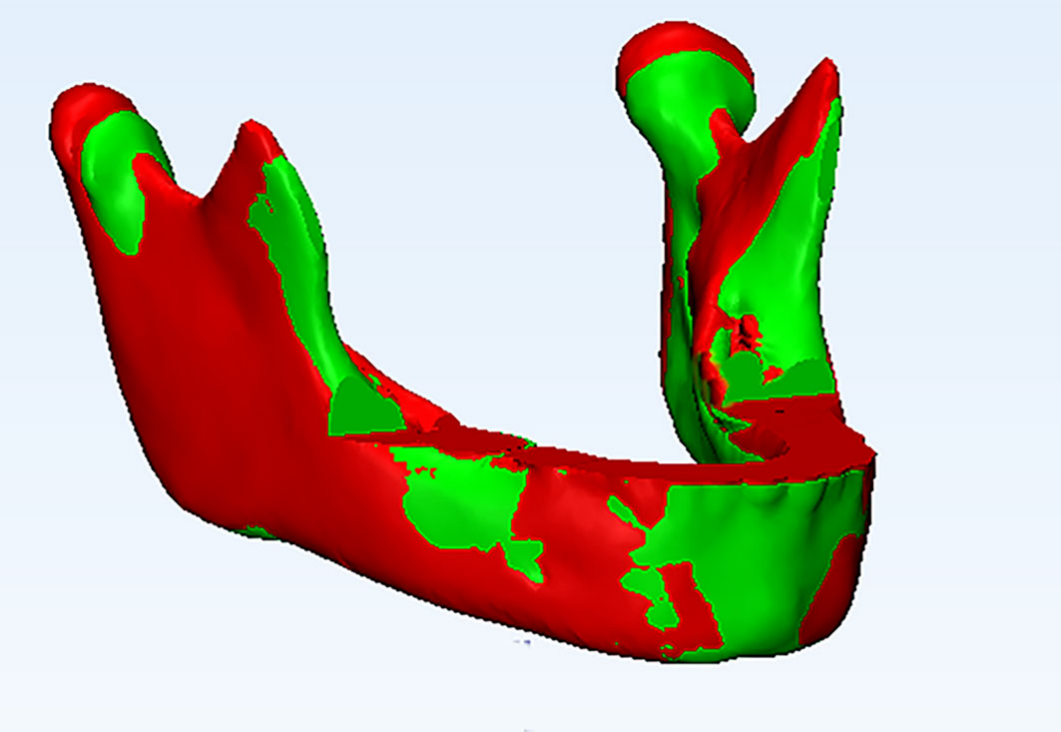
Isometric view of registered 3D mandibular models T1 (green mandible) and T2 (red mandible).

**Figure 5.**
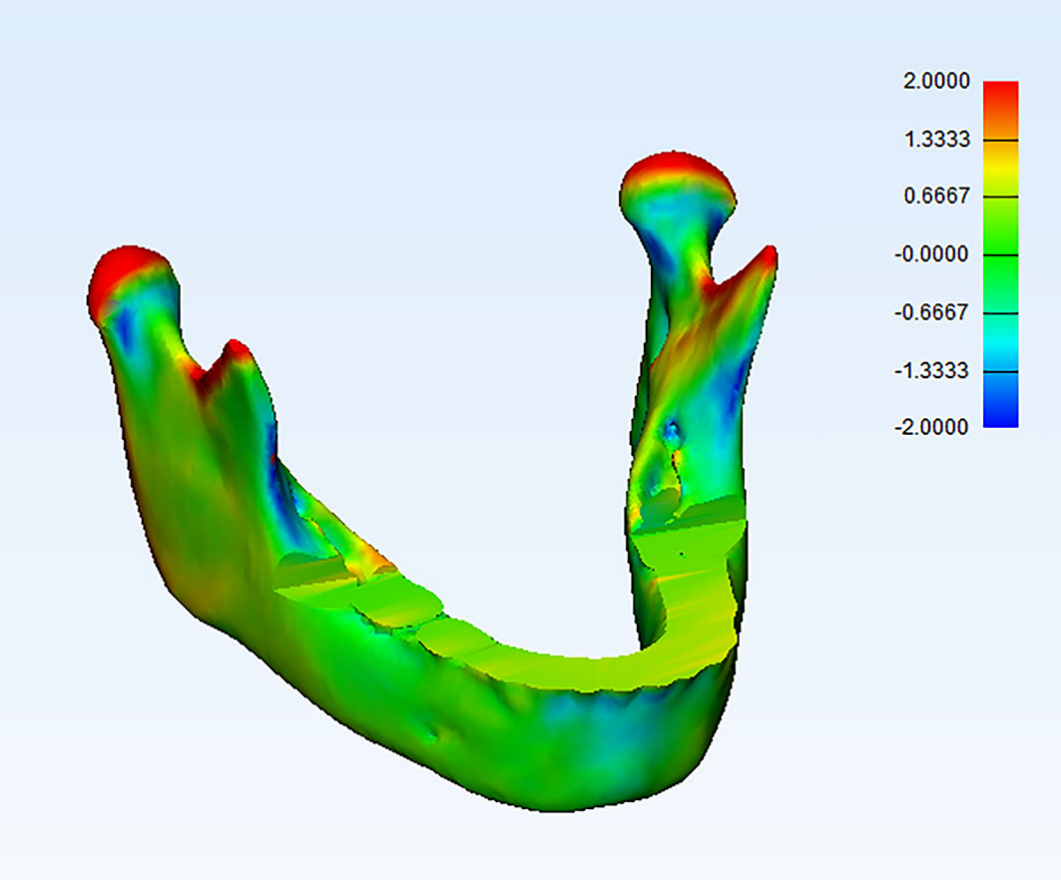
Isometric view of the registered 3D mandibular color-coded map after part analysis between T2-T1.

The paired resulting reconstructed mandibular models were exported to 3-Matic software (3-matic13.0, Materialise NV, Leuven, Belgium), and their surfaces were warped using the Warp tool of Mimics™. Initially, The T1 and T2 3D volumes were superimposed manually by approximating similar anatomical regions of the mandible using Interactive Translate and Rotate tools of the software, followed by automatic global registration in 3-Matic software ( Figure 4). The superimposition was repeated three times to enhance accuracy and reproducibility. The point-based analysis was performed to assess the changes in 3D mandibular models between T1 and T2, and a color map was produced to assess the mandibular shape changes. The threshold was set at 2 mm: green areas indicated differences within 2 mm (between −2 and 2 mm), red surfaces indicated positive values displacement more than 2 mm, and blue surfaces indicated negative values displacement greater than −2 mm between two 3D models ( Figure 5). Quantitative changes were done by reporting the mean, minimum, and maximum values of part analyses on a spreadsheet and used for comparative analyses

**Interim analyses and stopping guidelines**

No interim analyses were performed during this trial.

### Statistical analysis

Statistical analyses were performed using IBM SPSS Statistics for Windows, version 26.0 (IBM Corp., NY, USA). The average value of each bilateral measurement was calculated to achieve the statistics of this study. Shapiro–Wilk test was used to test the normality of data. Accordingly, non-normally distributed data were analyzed by the Wilcoxon signed-rank test and the Mann–Whitney U test for in- and intergroup comparison, respectively. Additionally, a paired-samples t-test was used to evaluate the changes in the treatment group, and an independent t-test was used for the intergroup comparisons of parameters with a normal distribution. Bonferroni's correction was used to adjust the alpha level due to multiplicity. The adjusted alpha level was 0.0125 for the volumetric and surface area measurements and 0.005 for the cephalometric analysis. To assess the reliability of the measurements, 17 (25%) images were randomly selected and re-measured after a 2-week interval by the same examiner (AH). Intraclass correlation coefficients (ICCs) with the absolute agreement were used to assess intra-rater reliability. Error of the method was analyzed with Dahlberg's formula. ^27^

## Results

### Reliability of the measurements

Intra-examiner reliability was high, and ICCs ranged from 0.83 to 0.999. The error of the method was acceptable for both linear and angular measurements, which were smaller than 0.5 mm and 0.6 degrees, respectively. The measurement error ranged from 18.84 to 54.3 mm ^3^ for the volumetric measurements and 18.81 to 44.58 mm ^2^ for the surface area measurements ( Table 3).

**Table 3.** Reliability of the performed measurements and error of the method.

Variable		95% confidence interval			Mean difference *	Error of the method **
		ICC	LB	UB		
Mandibular volume (mm ^3^)		0.993	0.981	0.998	17.19	54.3
Mandibular area size (mm ^2^)		0.995	0.986	0.998	17.92	44.58
Condyle volume (mm ^3^)		0.996	0.988	0.998	3.79	18.84
Condyle area size (mm ^2^)		0.973	0.925	0.990	3.31	18.81
Go-Gn (mm)		0.989	0.971	0.996	0.17	0.46
Co-Go (mm)		0.980	0.944	0.993	0.03	0.39
Co-Gn (mm)		0.994	0.984	0.998	0.23	0.43
Co-Go-Gn (degree)		0.950	0.982	0.993	0.22	0.6
CP-FH (mm)		0.945	0.847	0.980	0.14	0.37
CP-Y (mm)		0.964	0.900	0.987	0.13	0.41
FP-FH (mm)		0.941	0.838	0.979	0.18	0.27
FP- Y (mm)		0.980	0.945	0.993	0.07	0.39
AJS (mm)		0.967	0.908	0.988	0.05	0.11
SJS (mm)		0.982	0.950	0.993	0.08	0.24
PJS (mm)		0.998	0.993	0.999	0.18	0.26
Point-based analysis	Mean part analysis (mm)	0.932	0.823	0.975	0.01	0.09
	Minimum part analysis (mm)	0.998	0.996	0.999	-0.13	0.44
	Maximum part analysis (mm)	0.964	0.904	0.987	0.21	0.28

ICC: Intraclass correlation, LB: Lower bound, UB: Upper bound.

* Mean differences between the two measures.

** Evaluated using the Dahlberg's formula. ^27^

**Basic sample characteristics**

The CONSORT flow diagram of patients' recruitment and follow-up is given in Figure 1. In total, 38 patients (21 boys and 17 girls) were enrolled. ^44^ However, three patients were not included in the statistical analysis: one in each group was lost to follow-up, and the third one was extracted from the control group due to the low-quality low-dose CT image. Table 4 shows the basic sample characteristics. The mean treatment and observation periods were 16.67±0.52 months and 16.67±0.52 months, respectively. There were no significant differences in the age distribution and treatment/observation periods between the experimental and the control groups (p=0.675 and p=0.179, respectively).

**Table 4.** Baseline sample characteristics.

Variable	Control group (N=17)		Chin-cup group (N=18)		Mean difference	P-value	95% Confidence Interval of the Difference	
	Mean	SD	Mean	SD			Lower	Upper
Gender (female)	8 (47.05%)		8 (44.45%)			0.877 †	-	-
Age (year)	6.69	0.86	6.57	0.83	0.29	0.676 ^‡^	-0.70	0.46
Observation/treatment period (month)	16.67	0.52	17.02	0.92	0.12	0.179 ^‡^	-0.17	0.86

SD: Standard deviation.

^†^ Pearson's Chi-square test.

^‡^ Two-sample t-test.

### Outcome measures

The mean mandibular volume increased significantly by 777.24 mm ^3^ and 1,221.62 mm ^3^ in the chin cup and control groups, respectively. Also, the mean condylar volume increased significantly by 94.57 mm ^3^ and 132.54 mm ^3^ in the chin cup and control groups, respectively ( Table 5). The linear measurements of the mandible significantly increased in both groups (p<0.05). No statistically significant differences between the chin cup and control groups were observed regarding the volumes, superficial areas, and linear changes of the mandible and condyles ( Tables 5 and 6). Although the mean mandibular angle decreased by 1.63° in the treatment group, there was no statistically significant difference between the treatment and the control groups regarding this variable ( Table 6).

**Table 5.** Descriptive statistics of the area and volumetric changes that occurred in each group (T2-T1) as well as the p-values of significance tests.

	Control group (N=17)			Chin-cup group (N=18)			Control group vs Chin-cup group			
	Mean	SD	P value ^†^	Mean	SD	P value ^†^	Mean	P-value ^§^	95% Confidence Interval of the difference	
									Lower bound	Upper bound
Mandibular volume (mm ^3^)	1,221.62	641.52	<0.001 * ^‡^	777.24	797.42	0.002 * ^‡^	-444.38	0.110 ^‖^	-943.94	55.18
Mandibular area size (mm ^2^)	833.84	1,051.05	0.005 *	722.40	1,335.40	0.003 * ^‡^	-111.44	0.785	-941.21	718.33
Condyle volume (mm ^3^)	132.54	84.79	<0.001 * ^‡^	94.57	91.76	0.001 * ^‡^	-37.97	0.184 ^‖^	-98.83	22.89
Condyle area size (mm ^2^)	88.49	100.20	0.002 *	66.08	48.05	<0.001 *	-22.41	0.401	-75.96	31.14

Bonferroni's correction was applied to adjust the a level due to multiplicity. Adjusted alpha level was 0.0125.

SD: standard deviation.

† Paired t-test.

‡ Wilcoxon signed-rank test.

§ Two-sample t test.

^‖^ Mann-Whitney U test.

* Statistically significant at p<0.0125.

**Table 6.** Descriptive statistics of the cephalometric changes that occurred in each group (T2-T1) as well as the p-values of significance tests.

	Control group (N=17)			Chin-cup group (N=18)			Control group vs Chin-cup group			
	Mean	SD	P value ^†^	Mean	SD	P value ^†^	Mean	P-value ^§^	95% Confidence Interval of the difference	
									Lower bound	Upper bound
Go-Gn (mm)	1.64	2.00	0.004 * ^‡^	1.18	1.54	0.005 * ^‡^	-0.46	0.452 ^‖^	-1.68	0.77
Co-Go (mm)	1.49	1.60	0.003 ^‡^	1.05	1.57	0.014 ^‡^	-0.44	0.483 ^‖^	-1.53	0.65
Co-Gn (mm)	1.77	1.38	<0.001 *	1.53	2.15	0.008	-0.24	0.702	-1.49	1.01
Co-Go-Gn (degree)	-0.25	2.15	0.639	-1.63	2.80	0.024	-1.38	0.111	-3.11	0.34
CP-FH (mm)	-0.84	0.82	0.001 *	-1.68	0.75	<0.001 *	-0.84	0.003 *	-1.38	-0.30
CP-Y (mm)	-0.15	1.23	0.266 ^‡^	-1.29	1.03	0.001 * ^‡^	-1.14	0.003 * ^‖^	-1.92	-0.36
FP-FH (mm)	0.04	1.21	0.904 ^‡^	-0.81	0.51	<0.001 *	-0.91	0.004 * ^‖^	-1.53	-0.29
FP-Y (mm)	-0.11	0.77	0.557 ^‡^	-0.82	0.54	<0.001 * ^‡^	-0.71	0.004 * ^‖^	-1.14	-0.27
AJS (mm)	0.05	0.50	0.701	0.12	0.30	0.103	0.07	0.600	-0.21	0.36
SJS (mm)	0.10	0.54	0.309 ^‡^	-0.71	0.76	<0.001 * ^‡^	-0.81	0.017 ^‖^	-1.26	-0.36
PJS (mm)	0.07	0.35	0.421	-0.93	0.91	<0.001 *	-1.00	<0.001 *	-1.48	-0.52

Bonferroni's correction was applied to adjust the alpha level due to multiplicity. Adjusted alpha level was 0.005.

SD: standard deviation.

† Paired t-test.

‡ Wilcoxon signed-rank test.

§ Two-sample t test.

^‖^ Mann-Whitney U test.

* Statistically significant at p<0.005.

There was a significant posterior and superior displacement of the condyles following treatment by a mean of 1.29 mm (p=0.001) and 1.68 mm (p<0.001), respectively. Similar changes were observed in the glenoid fossa, but to a lesser extent by a mean of 0.82 mm posteriorly (p<0.001) and 0.81mm superiorly (p<0.001). The superior joint space (SJS) and posterior joint space (PJS) showed a significant mean decrease of 0.71 mm and 0.93 mm, respectively. However, no statistically significant differences were observed in the relative positions of the condyles, glenoid fossa, and joint spaces in the control group (p>0.05), except for a significantly superior displacement of the condyles (0.84 mm; p=0.001). The changes in the relative sagittal and vertical positions of condyles and glenoid fossa were significantly smaller in the treatment group than in the control group. No statistically significant differences were observed between the treatment and control groups regarding the average mean, maximum, and minimum part analysis of the point-based analysis (p>0.05; Table 7).

**Table 7.** Descriptive statistics of quantitative displacement parameters of the superimposed 3D models (T2-T1) as well as the p-values of significance tests.

		Control group (N=17)		Chin-cup group (N=18)		Control vs. Chin-cup		95% Confidence Interval of the Difference Control vs. chin-cup	
		Mean	SD	Mean	SD	Mean	P-value †	Lower	Upper
Point-based analysis	Mean part analysis (mm)	0.20	0.31	0.07	0.34	0.13	0.143	-0.09	0.36
	Minimum part analysis (mm)	-2.26	1.08	-2.97	1.38	-0.71	0.076 ‡	-1.15	1.56
	Maximum part analysis (mm)	2.59	1.61	2.20	1.36	-0.39	0.463	-0.63	1.41

SD: standard deviation.

† Mann-Whitney U test.

‡ Two-sample t–test.

**Harms**

No serious harm was observed.

## Discussion

Although the extensive literature on the potential condyle-mandibular adaptation of the chin cup is of interest, the conflicting outcomes urge high-quality studies that apply 3D imaging technology to the study of the TMJ, such as CBCT or CT scan. ^1^^,^^2^^,^^12^ But, the difficulty achieving condylar segmentation in CBCT images is due to its lesser density compared to the rest of the mandible, as demonstrated previously. ^28^ Therefore, the use of low-dose CT imaging was preferred in this study. In addition, the acceptable radiation dose of this imaging method is 0.51 mSv under the acceptable limit of 1 mSv per year. ^13^^,^^15^

Precise volumetric assessment can be affected by several variables, such as voxel size, patient position, and the characteristics of the software used for segmentation. ^28^^,^^29^ The used software has been proven by Weissheimer ^30^ as the most accurate software with errors in the volume segmentations less than 2% of the real size.

The influence of sex on the treatment outcomes was not accounted for in this study. Baccetti ^31^ found no sex differences in most dentofacial parameters for Class III patients until the age of 13 years. The mean age of the treatment group was 6.59 (±0.86) years, which has been considered early optimal treatment timing. ^32^ Additionally, the mean age of the control group sample was 6.57 (±0.83) years, which gives the patients the time to be treated ideally after the observation period. The treatment period was 17.02 (±0.92) months. Mitani ^33^ stated that the major treatment effects of the chin cup are observed in the first 2 to 3 years of treatment. Some previous studies suggested variable treatment periods from 1-7 years, but most stopped heavy forces after obtaining a good overjet. On the other hand, treatment effects can be better demonstrated if longer periods of observation were implemented in the current study.

Although the force applied from the chin cup, the volume and the superficial area of the mandible showed a significant increase. These changes could be secondary to simultaneous bone repositioning on most surfaces of the mandible. ^34^ Similarly, the linear mandibular measurements increased significantly in both groups. It was observed that the mean of the increase in the mandibular dimensions was smaller in the treatment group than in the untreated group, but no differences were found between both groups regarding these parameters. Due to several studies on chin cup therapy using 2D measurements based on cephalometric radiographs, only linear measurements of this trial could be compared. The results of this study regarding the changes in the linear mandibular measurements were in agreement with some relevant clinical studies. ^6^^–^^8^ On the contrary, Mimura and Deguchi ^35^ reported a similar increase in the mandibular length in the chin cup and control groups; these data suggest that chin cup use does not decrease the overall mandibular growth. As mentioned previously, the differences in the appliance design, the force application, and the evaluation method might cause differences in the results.

The gonial angle showed a closing pattern at the end of treatment. This gonial angle change has also been reported in the literature. ^7^^,^^8^^,^^36^ Lin *et al*. ^37^ attributed the reason to the force's direction that tends the mandible's ramus to rotate around the gonial angle. ^34^ Nevertheless, the observed change in the gonial angle in this trial was insignificant in the chin cup group and among the groups. It is more likely to be affected by the individual growth pattern, as the gonial area has some freedom to remodel. ^8^

The condyle's volume and superficial area increased significantly in both groups. It is important to highlight that even though no statistically significant differences were found, the overall changes in the condyles in the treatment group were smaller than in the control group. This change had been previously demonstrated by experimental studies showing a decrease in the condylar growth rate due to the compression force applied by the chin cup. ^18^

Another point that should be considered is the observed extensive standard deviation in the volumetric condylar change. The individual differences in the condyles' size, form, and growth cartilage; thus, growth magnitude should also be individually different. In turn, the chin cup force affects condyles variably in the clinical environment. ^33^

The distinguishing differences between the two groups involved most of the TMJ parts. The condyle was displaced posteriorly and superiorly in the chin cup group and accompanied by a posterior and posterior remodeling of the glenoid fossa. However, it showed lesser superior and posterior displacement than the condyle. The histological differences between the glenoid fossa and the condyles could be the reason for the non-equivalent changes. ^38^ Interestingly, Pancherz *et al*. ^39^ observed a later remodeling response of the glenoid fossa than that occurring in condyles after the Herbst appliance. The study findings regarding the condyles and glenoid fossa portions were similar to those of Mimura and Deguchi. ^35^ However, neither the amounts of displacement nor the evaluation method was similar. On the contrary, Gökalp and Kurt ^40^ showed no changes in condylar positioning by magnetic resonance imaging after chin cup therapy. The evaluation method, however, might cause differences in the results.

In this context, Lee *et al*. ^41^ found that the facemask appliance induced similar effects but with a smaller magnitude. Results from Grandori ^42^ could explain the difference, revealing that only 70% of the facemask force was translated to the chin. Surprisingly, De Clerk *et al*. ^43^ used bone-anchored Class III intermaxillary traction with a force of 250 g/side, but the amounts of displacement of the condyle and glenoid fossa were greater than those observed in this study. These differences might be due to the duration of the applied force and the age of the sample in his study, which was older and closer to puberty than in this study.

The results indicated a decrease in the spaces of the joint, except for the anterior space. This may be attributed to the backward and upward forces applied from the chin cup, with the slow remodeling of the glenoid fossa, as mentioned previously. ^39^ Gökalp and Kurt’s ^40^ study showed no change in the condyle-glenoid fossa relationship. However, the evaluation method and the relatively small sample size make the comparison meaningless. These changes require long-term evaluation to corroborate the TMJ adaptation to these positional changes.

**Figure 6.**
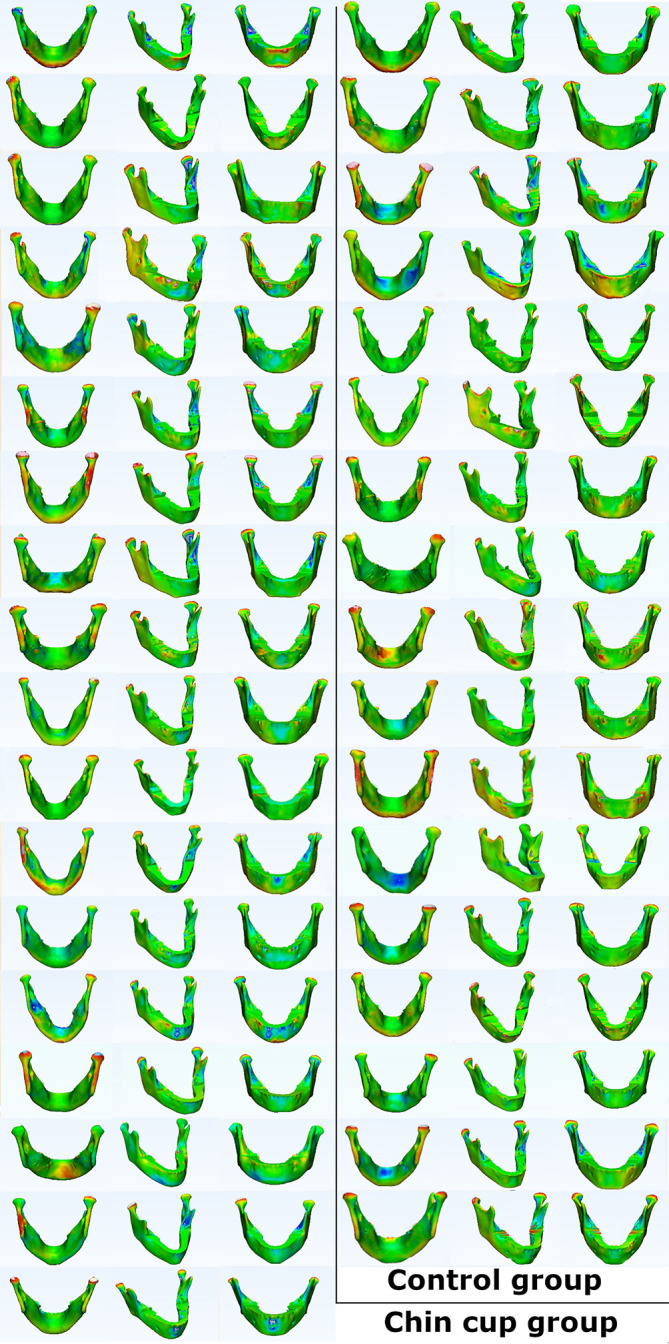
Frontal, isometric and back views of point-based analysis color map.

The analysis of the changes in mandibular shape through 3D superimposition showed no differences between the two groups, except some inward changes were noticed in the symphysis region, which is indicated by the blue areas in the color mapping in Figure 6. This type of change has been reported previously and might be induced by the plastic cup of chin cup. ^8^^,^^33^ The condyles showed atypical changes. On the contrary, the past 2D cephalometric studies assumed that chin cup treatment could induce a forward bending of the condylar neck. ^8^^,^^35^ The individual differences in growth direction and magnitude, and sometimes the asymmetric position of the condyles, could affect condylar reaction either to the chin cup or the growth. ^33^

Quantitative results of the point-based analysis showed positive mean displacement in both groups. The average mean and maximum point part analysis was smaller in the chin cup group than in the control group. Although intergroup comparisons according to the parameters of the point part analysis showed insignificant differences between the two groups, class III malocclusion worsens in untreated subjects over time; thus, early treatment is required.

### Limitations

One of the limitations of the current trial is its short-term evaluation. Secondly, the temporomandibular disk was not studied in this trial. However, this aim needs another imaging method.

### Generalizability

Randomized controlled trials (RCTs) provide the highest level of evidence. ^10^ Additionally, three-dimensional quantification gives a true mandibular, condyle, and glenoid fossa changes after orthodontic treatment by the chin cup. ^43^ Moreover, significant changes occur in the craniofacial region during the mixed dentition that can be utilized for orthodontic therapy. ^32^ Our study designed as RCT with the help of the three-dimensional quantification and patients were in the early mixed dentation, as a result, the current research findings revealing the effects of the chincup are generalizable. In this randomized controlled trial, the chin cup treatment failed to control mandibular growth, resulting only in orientation to condylar positions and compression of joint spaces.

## Conclusions

In conclusion, the chin cup seemed to have no effects on mandibular dimensions in this study. The major action of the retroactive force of the chin cup oriented onto the condylar postions and was associated with compression of the joint spaces.

## Data Availability

Figshare: Evaluation of the Dimensional Changes in the Mandible, Condyles, and the Temporomandibular Joint Following Skeletal Class III Treatment with Chin Cup and Bonded Maxillary Bite Block Using Low-Dose Computed Tomography: A Single-center, Randomized Controlled Trial. https://doi.org/10.6084/m9.figshare.21973517.v3. ^44^ This project contains the following underlying data:
- Data file 1: linear anlaysis.xlsx - Data file 2: part analysis.xlsx - Date file 3: volume and area analysis.xlsx Figshare: Evaluation of the Dimensional Changes in the Mandible, Condyles, and the Temporomandibular Joint Following Skeletal Class III Treatment with Chin Cup and Bonded Maxillary Bite Block Using Low-Dose Computed Tomography: A Single-center, Randomized Controlled Trial. https://doi.org/10.6084/m9.figshare.21973517.v3. ^44^ This project contains the following extended data:
- Extended data file 1: Information sheet (in Arabic and English) - Extended data fille 2: Consent form (in Arabic and English) Figshare: CONSORT checklist for ‘Evaluation of the Dimensional Changes in the Mandible, Condyles, and the Temporomandibular Joint Following Skeletal Class III Treatment with Chin Cup and Bonded Maxillary Bite Block Using Low-Dose Computed Tomography: A Single-center, Randomized Controlled Trial’. https://doi.org/10.6084/m9.figshare.21973517.v3. ^44^ Data are available under the terms of the Creative Commons Zero “No rights reserved” data waiver (CC0 1.0 Public domain dedication).
