# Supplementary material for: Evaluation of the dimensional changes in the mandible, condyles, and the temporomandibular joint following skeletal class III treatment with chin cup and bonded maxillary bite block using low-dose computed tomography: A single-center, randomized controlled trial
Source: F1000Res. 2023 Mar 13;12:264. [Version 1] doi: 10.12688/f1000research.130941.1 (PMC10050907; doi:10.12688/f1000research.130941.1)
Supplement: Peer review history

# Peer review: Reviewer response for version 1

**DOI:** 10.5256/f1000research.143739.r166187

**Published:** 2023-04-11

**Authors:** Rozita Hassan

**Affiliations:** 1 Department of Orthodontics, School of Dental Sciences, Universiti Sains Malaysia, Minden Heights, Pulau Pinang, Malaysia

The manuscript was overall well-written with good command of English. It is a clinical trial and utilized an advanced method of measurement technology.

 The finding helps the clinician to understand more about the outcome of Class III malocclusion patients treated with a chin cup.

 However one of the radiological inclusion was 0< ANB>4 which indicated inclusive of Skeletal Class 1. Perhaps the title needs to be revised. My suggestion is to change to Class III malocclusion rather than Skeletal class III. 

 In a view of the chin cup type, there is a lacking of information regarding the design and company brand.

Is the work clearly and accurately presented and does it cite the current literature?

Yes

If applicable, is the statistical analysis and its interpretation appropriate?

Yes

Are all the source data underlying the results available to ensure full reproducibility?

Yes

Is the study design appropriate and is the work technically sound?

Yes

Are the conclusions drawn adequately supported by the results?

Yes

Are sufficient details of methods and analysis provided to allow replication by others?

Yes

Reviewer Expertise:

Orthodontist

I confirm that I have read this submission and believe that I have an appropriate level of expertise to confirm that it is of an acceptable scientific standard.

# Peer review: Reviewer response for version 1

**DOI:** 10.5256/f1000research.143739.r166185

**Published:** 2023-04-11

**Authors:** Ahmad Hamdan

**Affiliations:** 1 Department of Orthodontics and Pediatric Dentistry, School of Dentistry, The University of Jordan, Amman, Amman Governorate, Jordan

Thanks you for asking me to review this interesting paper.

 This is a well designed study with an adequate sample size calculation and randomization. The methodology is sound and I am pleased that a reproducibility study was carried out, Although I would have liked to see a paired sample T test performed between repeat measurement to determine random error. 

 Statistical analyses could benefit from multiple variable comparisons using ANOVA since changes in many of the variable are related,

 The results are well presented and illustrated and based on the gathered data.

 Discussion is adequate and analyses all the main results. 

 My main concerns are related to the high standard deviations of many of the variable which is is an indication that the data should be interpreted with caution. In addition, the influence of the bite block without chin cup is not discussed.

Is the work clearly and accurately presented and does it cite the current literature?

Yes

If applicable, is the statistical analysis and its interpretation appropriate?

Yes

Are all the source data underlying the results available to ensure full reproducibility?

Yes

Is the study design appropriate and is the work technically sound?

Yes

Are the conclusions drawn adequately supported by the results?

Yes

Are sufficient details of methods and analysis provided to allow replication by others?

Yes

Reviewer Expertise:

Orthodontics

I confirm that I have read this submission and believe that I have an appropriate level of expertise to confirm that it is of an acceptable scientific standard, however I have significant reservations, as outlined above.

# Peer review: Reviewer response for version 1

**DOI:** 10.5256/f1000research.143739.r166188

**Published:** 2023-03-28

**Authors:** Mohammad Khursheed Alam

**Affiliations:** 1 Orthodontic Division, Preventive Dentistry Department, College of Dentistry, Jouf University, Sakaka, Saudi Arabia

Thank you very much for inviting me to review this manuscript. The study aimed to evaluate the dimensional changes in the mandible and the condyles following Class III treatment in growing patients. Orthopedic forces were applied to study the post-treatment changes. An RCT design was adopted to answer the research question. A control group of untreated patients was used to filter out the changes that may occur during the observation period due to growth. The study included 38 patients who were distributed equally into the two groups (with 19 patients in each group). The method of assessment was based on low-dose CT images which were taken twice (before and after the active treatment).

 I think that this study is well-conducted and well-written, and it delivers important information regarding the dimensional changes of the condyles, glenoid fossae, and the body of the mandible after treating the chin cup in treating children with prognathic mandibles. The research question is clearly stated. The materials and methods section contains detailed information about the different steps in conducting this work. The results are displayed using good tables and figures. An in-depth discussion is given in this manuscript, and it seems the authors are well familiar with recently published papers on this topic.

**Major points**

 After careful reading of this paper, I don't see major points that require an author's response.

**Minor points**

 The following few points require the author's attention

- In the Abstract section, the authors say, "Low-dose CT images were acquired before (T1) and after achieving (2-4 mm) positive overjet (T2), and after 16 months apart in both groups". According to my understanding from the paper, the sentence should be changed to appear as follows "Low-dose CT images were acquired before treatment (T1) and after achieving 2 to 4 mm positive overjet with at least 16 months of active treatment (T2)."
- In the Materials and Methods section, the authors say, "The radiological inclusion criteria were (1) mild to moderate mandibular prognathism (SNB > 80°, and 0 ≤ANB≥4)". Please correct this condition since the angle "ANB" should lay between 0 and – 4 (minus 4).
- In the Materials and Methods section, the authors say that they used the ANB angle to determine Class III patients who should be enrolled in the study. If "Witz appraisal" was used to confirm the diagnosis, please indicate that in the text.
- Under the "Interventions" subheading, the appliance was written "Chin-cup". In other places in this manuscript, it appears as "chin cup". Please try to mention this appliance in a standardized manner, i.e., either "chip-cup" or "chin cup", throughout the whole manuscript.
- In the "Interventional group" subheading, please use the term "retraction force" instead of "traction force".
- Under the "Outcome measures" subheading, the authors say that patients were seated in a supine position with their Frankfurt Horizontal (FH) plane perpendicular to the floor. This information is wrong. Their Frankfurt Horizontal plane should be parallel to the floor. Please correct.
- Under the "The mandible and condyles reconstruction" subheading, please replace the term "surface sizes" with "surface areas".
- In the "Statistical analysis" subsection, please add the word "distributions" at the end of this sentence "Shapiro-Wilk test was used to evaluate the Noramilty of data distributions".
- In the "Hrams" subheading, please provide any information about symptoms related to the TMJ disorders that may have developed in the experimental group (the chin cup group). If you encountered no adverse effects on the TMJs, please declare this result in this section.
- In the "Limitations" subheading (in the Discussion section), please add the limitation regarding the absence of any objective clinical examination of TMJ disorders after the chin cup therapy. You have already shown that there was compression in the joint spaces. Therefore, one could assume that problems may have arisen in the TMJ regions.

Overall, the paper is well-written, and the authors should be congratulated for their great research work. The clinical message is clear from this RCT.

Is the work clearly and accurately presented and does it cite the current literature?

Yes

If applicable, is the statistical analysis and its interpretation appropriate?

Yes

Are all the source data underlying the results available to ensure full reproducibility?

Yes

Is the study design appropriate and is the work technically sound?

Yes

Are the conclusions drawn adequately supported by the results?

Yes

Are sufficient details of methods and analysis provided to allow replication by others?

Yes

Reviewer Expertise:

NA

I confirm that I have read this submission and believe that I have an appropriate level of expertise to confirm that it is of an acceptable scientific standard.

# Peer review: Reviewer response for version 1

**DOI:** 10.5256/f1000research.143739.r166186

**Published:** 2023-03-23

**Authors:** Ghassan Idris

**Affiliations:** 1 School of Medicine and Dentistry, Griffith University, Gold Coast, Queensland, Australia

Evaluation of the dimensional changes in the mandible, condyles, and the temporomandibular joint following skeletal class III treatment with chin cup and bonded maxillary bite block using low-dose computed tomography: A single-center, randomized controlled trial

 The study is a randomized controlled trial on 38 Class III growing children. The study used the chin cup combined with maxillary bonded posterior bite planes. Volumetric and two- dimensional linear assessment were carried out using CBCT in two times. Before starting the treatment and after achieving psotive overjet in the treatment group and in 16 months in the control group.

 The report is well written and answering an important question to fill a gap in the literature regarding the use of Chin Cup which were always debatable among orthodontists. The current repot is thorough following the CONSORT guidelines and the outcome and conclusion is related to the reported data which is satisfactory.

 One point I feel it is a bit confusing and it needs to be clarified is the follow up times. It is clear that the follow ups were at T1 and T2 however the following sentence in the Abstract section “ Low-dose CT images were acquired before (T1) and after achieving  (2-4 mm) positive overjet (T2), and after 16 months apart in both groups.” Is giving the impression that there were a separate third time follow up at 16 months. Please, this sentence needs rephrasing.

 Congratulations for authors on the good work and well written report.

Is the work clearly and accurately presented and does it cite the current literature?

Yes

If applicable, is the statistical analysis and its interpretation appropriate?

Yes

Are all the source data underlying the results available to ensure full reproducibility?

Yes

Is the study design appropriate and is the work technically sound?

Yes

Are the conclusions drawn adequately supported by the results?

Yes

Are sufficient details of methods and analysis provided to allow replication by others?

Yes

Reviewer Expertise:

Orthodontics, Craniofacial anomalies and Cleft Lip and Palate Children, Functional appliances in growing children, Sleep Apnoea

I confirm that I have read this submission and believe that I have an appropriate level of expertise to confirm that it is of an acceptable scientific standard.
